# Standardized Phase Angle for Predicting Nutritional Status of Hemodialysis Patients in the Early Period After Deceased Donor Kidney Transplantation

**DOI:** 10.3389/fnut.2022.803002

**Published:** 2022-02-16

**Authors:** Diana Sukackiene, Laurynas Rimsevicius, Marius Miglinas

**Affiliations:** Institute of Clinical Medicine, Faculty of Medicine, Vilnius University, Vilnius, Lithuania

**Keywords:** phase angle (PA), kidney transplantation, bioelectrical impedance analysis (BIA), z-score, handgrip strength (HGS)

## Abstract

**Background:**

This study was designed to verify whether early posttransplant standardized phase angle (SPhA) determines nutrition status of hemodialysis patients in regard to different nutritional markers and predicts handgrip strength (HGS) 6 months after kidney transplantation.

**Methods:**

A total of 82 kidney transplant recipients on maintenance hemodialysis treatment entered the study. Nutritional status was evaluated before kidney transplantation, at the hospital discharge date, and 6 months after. We used bioelectrical impedance analysis (BIA), three different malnutrition screening tools, HGS, and anthropometric measurements. Demographic profiles and biochemical nutritional markers were collected. SPhA values, adjusted for age and BMI, were used in our study.

**Results:**

In the early posttransplant period, kidney transplant recipients lost muscle mass, gained fat mass, and developed mostly negative SPhA, accompanied by significantly lower albumin levels. The subjects with lower than median (<-1.46) SPhA_dis_ [the SPhA (at discharge) adjusted for hospitalization time and the baseline SPhA] displayed lower values of albumin concentration (43.4 vs. 45.1 g/l, *p* = 0.010), hemoglobin (124 vs. 133 g/l, *p* = 0.016), GNRI (113 vs. 118, *p* = 0.041), and HGS (30 vs. 33 kg, *p* = 0.043). These patients had higher ferritin concentrations (420 vs. 258 mkmol/l, *p* = 0.026), longer inpatient stays (32 vs. 21 days, *p* < 0.001), and higher MIS scores (3 vs. 1, *p* = 0.001).

**Conclusion:**

At the moment of hospital discharge, lower than the median SPhA is related to protein-energy wasting, represented as lower concentrations of nutrition biomarkers and an active inflammatory response. Higher SPhA before kidney transplantation predicts HGS 6 months after kidney transplantation, especially in women.

## Introduction

Kidney transplantation is considered as the optimal treatment option for end-stage kidney disease. It enables greater longevity and better quality of life compared to peritoneal dialysis and hemodialysis (1, 2). The early posttransplant (the first 6 months) period carries a risk of significant complications, which includes those related to nutrition and metabolic changes (3). In simple terms, kidney transplant recipients could be considered both “gainers” and “losers” by reflecting different posttransplant shifts within body compartments.

During the last decade, nutritional status is shown to be a relevant clinical factor in patients with chronic kidney disease (CKD), as malnutrition and sarcopenia are strongly associated with prolonged hospitalization, increased risk of complications, infections, morbidity, and mortality (4). Kidney transplant recipients may also suffer from the loss of muscle mass that is replaced by fat mass. These changes might be caused by the following triggers: low physical activity, uremic toxins, inflammation, urine and/or dialysate nutrient losses, catabolic and anabolic hormone dysfunction, and metabolic acidosis (5, 6).

A number of nutrition assessment tools are available on the market. One of them is bioelectrical impedance analysis (BIA). BIA has been advocated as a simple, safe, and non-invasive technique. It measures body composition by determining the resistance and reactance of the body against an alternating electrical current (5). The measurement of phase angle (PhA) is the most clinically established BIA parameter and has been interpreted as an indicator of membrane integrity and body cell mass (7). It has been acknowledged as a valuable measurement for nutritional assessment and as an important predictor of health status in a variety of diseases (8).

Currently, a limited number of studies have investigated PhA measurement importance in kidney transplant recipients. To our knowledge, none of them provided standardized PhA values for either CKD population or for patients after kidney transplant. Therefore, in this study, we aimed to verify whether standardized PhA (SPhA) measured during the early posttransplant—at the discharge from the hospital and then after 6 months of follow-up—reflects on other compartments of body composition, biochemical nutritional markers, and handgrip strength (HGS).

## Subjects and Methods

### Study Design and Patients

A longitudinal observational study was conducted in a tertiary referral university hospital between January 2018 and March 2020. The aim of this study was to measure BIA-derived PhA and to investigate its associations with other BIA-derived nutritional parameters: fat mass (FM), fat-free mass (FFM), skeletal lean mass (SLM), skeletal muscle mass (SMM), HGS, patient demographics, and biochemical markers at the baseline (before KT), at the discharge day and 6 months posttransplant.

The eligibility criteria were as follows: (1) undergoing hemodialysis (HD) > 3 months, (2) age ≥ 18 years, and (3) signed informed consent form. Exclusion criteria included limbless patients, pacemakers, and patients who refused to participate in the study.

In our hospital, the recipients after kidney transplant surgery are transferred to the intensive care unit for 24–48 h, and then, they are moved to the urology department for a week and the rest of time they recover in the kidney transplantation department. The patients remain in the hospital for a total of about 14–21 days. Dietary therapy during recovery period consists of high protein, fiber, and low salt intake.

The flow chart of patient selection.

**Figure:**
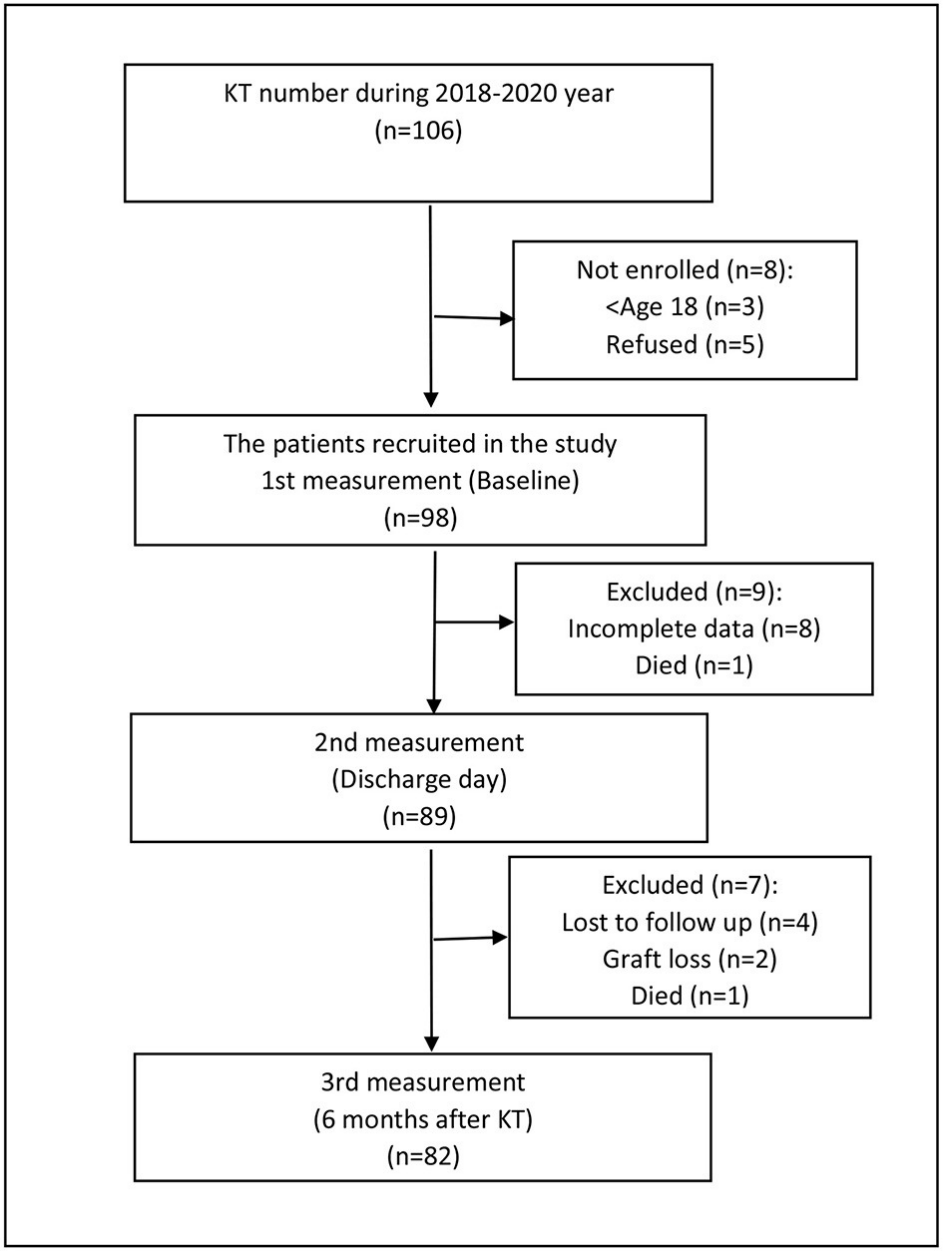


### Ethics

Permissions from the regional research ethics committee (approval number 158200-17-972-470) and the research ethics committee of the hospital were acquired. Informed consent was obtained from all patients prior to enrollment in the study. The study was conducted in accordance with the principles of Declaration of Helsinki.

### Laboratory Data

Two times during the course of the study [at the baseline (before KT) and 6 months later], blood samples were collected to assess the following parameters: serum albumin, prealbumin, ferritin, transferrin, and high-sensitivity C-reactive protein (hs-CRP). The blood samples were collected into BD Vacutainer SST-II Advance Serum Separator Tubes (BD Diagnostics, UK) by venipuncture from all participants after a 12-h overnight fasting period. Samples for all biochemical markers were centrifuged at the local clinical chemistry laboratory (3230 RCF, ambient temperature, 7 min) and analyzed the same day using standard automated methods. The CKD-EPI formula was used for estimating glomerular filtration rate (eGFR).

### Evaluation of Nutritional Status

#### Anthropometric Data

A trained nephrologist performed the anthropometric measurements for all participants. Height (cm) and weight (kg) were measured using an automatic scale with a sensitivity of 0.1 cm and a resolution of 0.1 kg. BMI was calculated as a ratio between weight and height in meters squared (kg/m^2^). Waist and hip circumferences (cm) were examined using a measuring tape, and the waist-to-hip ratio was calculated.

#### Handgrip Strength Assessment

Handgrip strength was measured using a Saehan hydraulic hand dynamometer (Model SH5002) with a scale of strength up to 100 kg. HGS was evaluated on the non-fistula dialysis arm or, if there was no fistula, on the dominant arm, as the arteriovenous fistula (AVF) is usually located in the non-dominant arm (9). Three measurements were taken with an interval of 5 s between measurements, and the highest value was used for analysis.

#### Subjective Global Assessment and Malnutrition Inflammation Score

The 2020 Updated Clinical Practice Guideline for Nutrition in CKD published by the National Kidney Foundation/Kidney Disease Outcome Quality Initiative recommended the 7-point subjective global assessment (SGA) (7p-SGA) and malnutrition inflammation score (MIS) for the assessment of nutritional status in patients with CKD on HD. We have chosen to use SGA and MIS, which were administered through face-to-face interviews and have been previously reported to be applicable tools in this population (10).

The SGA scores patients on a scale ranging; A—well nourished, B—mild-to-moderately malnourished, C—severely malnourished (11).

For the MIS, the cutoff proposed by Yamada et al. was used to classify the nutritional status: 0 to 5—well-nourished; 6 to 10—mild protein-energy wasting (PEW), and ≥11—moderate-to-severe PEW (12).

#### Phase Angle Measurement

Bioelectrical impedance analysis was performed to obtain the measurements of resistance (R) and reactance (Xc) using a calibrated body composition analyzer (InBody S10, Biospace, Seoul, Korea), which applies a single frequency of 50 kHz. The analysis was conducted for patients in a reclining posture and according to all recommendations from ESPEN and the manufacturers (13).

The PhA was calculated automatically by the BIA device from these two components according to the following formula: phase angle (°) = (reactance/resistance) × (180°/π) (8).

Interestingly, many authors focus on the calculation of a standardized phase angle (SPhA), which aims to account for confounding factors when determining PhA. An SPhA is calculated as a z-score that may be based on established population reference values and allows assessment of individual deviations from age-, sex-, and BMI-specific population (14). We used standardized PhA values adjusted for age and BMI acquired from Bosy-Westphal et al. to calculate PhA z-scores (15). In summary, we calculated PhA z-scores as follows:


$$\begin{array}{l} \text{SPhA = }[\text{PhA }(\text{study population})\\ \;-\text{reference PhA value}]\text{/reference}\;\text{SD} \end{array}$$

SD, standard deviation.

Then, the SPhA (at discharge) was adjusted for hospitalization time and the baseline SPhA and labeled as the “SPhA_dis_.”

PhA and SPhA values for each study patient are provided in the Supplementary Material.

### Statistical Analysis

Data were analyzed using R commander (Rcmdr) version 3.3.2. Continuous variables are expressed as the mean ± standard deviation (SD), discrete variables as medians with min–max values in parentheses, and categorical variables as percentages. To check the equality of two populations, an F-test and, if appropriate, Student's *t*-test or a two-sample Wilcoxon test were applied, and for categorical variables, a chi-square test was used. Multivariate linear regression analysis was used to evaluate potency of SPhA at the baseline and at discharge to predict HGS 6 months after kidney transplantation following corrections for age, sex, and baseline HGS. Since in men, these associations were absent, and we reported only data on women.

*p*-values lower than 0.05 were considered as statistically significant.

## Results

### Patient Characteristics

We enrolled 82 kidney transplant patients who received standard immunosuppressive treatment, which includes methylprednisolone, calcineurin inhibitor, and mofetil mycophenolate, after deceased-donor kidney transplantation. Baseline characteristics are presented in Table 1. The underlying kidney disease encompasses glomerulonephritis, polycystic kidney disease, inherited diseases, diabetes nephropathy, amyloidosis, pyelonephritis, kidney stones, and postrenal pathologies. The mean cold ischemia time was 14 h 30 min, and 63% (52) experienced delayed graft function but without acute transplant rejection.

**Table 1:** Baseline characteristics of kidney transplant recipients.

**Variable**	***N* = 82**
Age, year	43.9 (12.9)
Women, yes	43 (35)
Diabetes, yes	11 (9)
Residual kidney function, yes	55 (45)
Inpatient treatment duration, days	26 (12)
Immunosuppression	Tacrolimus 95 (78) MMF 100 (82) Cyclosporin 5 (4)
SGA	A 59 (48) B 41 (34)
GNRI score	114 (10)
MIS score	5 (2)

*Data expressed as mean (SD) or percent (number of patients)*.

*SGA, subjective global assessment; GNRI, geriatric nutritional risk index; MIS, malnutrition inflammation score*.

### Nutrition Status Change During Early Posttransplant Period

The prevalence of protein-energy wasting (PEW) before kidney transplantation was 44% and decreased to 7% after surgery. We compared nutrition-related variables during early posttransplant period (Table 2), that is, at the baseline (before transplantation)—labeled as “basal,” on the hospital discharge day—labeled as “dis,” and 6 months after kidney transplantation—labeled as “6 mo.” During the in-hospital stay, the study subjects had lost muscle mass, but gained fat mass instead and developed mostly negative SPhA_dis_ accompanied by significantly lower plasma albumin levels.

**Table 2:** Comparison of nutrition-related variables during the early posttransplant period.

**Variable**	**“basal”**	**“dis”**	**“6 mo”**	***p*-value “basal” vs. “dis”**	***p*-value “basal” vs. “6 mo”**
Weight, kg	78 (18)	75 (18)	78 (18)	<0.001	0.52
BMI, kg/m^2^	25.9 (5.2)	24.8 (4.9)	26.1 (5.2)	<0.001	0.46
Body fat percent	21 (12)	23 (11)	25 (11)	0.003	<0.001
Fat-free mass, kg	61 (15)	57 (13)	58 (13)	<0.001	<0.001
Fat mass, kg	17 (12)	18 (11)	21 (12)	0.042	<0.001
Fat-free mass index, kg/m^2^	20 (3)	19 (3)	19 (3)	<0.001	<0.001
Fat mass index, kg/m^2^	6 (4)	6 (4)	7 (4)	0.048	<0.001
Muscle mass, kg	34 (9)	31 (7)	32 (7)	<0.001	<0.001
Muscle mass index, kg/m^2^	11 (2)	10 (2)	10 (2)	<0.001	<0.001
BCM, kg	40 (10)	37 (8)	37 (8)	<0.001	<0.001
SPhA, °	−0.28 (1.65)	−1.50 ^*^(1.23)	−1.46 (1.16)	<0.001	<0.001
Positive SPhA, yes	37 (30)	10 (8)	7 (6)	<0.001	<0.001
TBW, L	45 (11)	42 (9)	43 (9)	<0.001	<0.001
ECW, L	17 (4)	16 (5)	17 (4)	0.029	0.137
ICW, L	27 (7)	26 (6)	25 (6)	<0.001	<0.001
Albumin, g/l	44 (4)	38 (1)	44 (4)	<0.001	0.63
Handgrip strength, kg	37.8 (13.6)	–	32.7 (12.1)	–	<0.001

*Data expressed as mean (SD) or percent (number of patients)*.

* *Unadjusted*.

*BMI, body mass index; SPhA, standardized phase angle (PhA z-score); positive SPhA, the raw PhA score is higher than the mean average score presented by Bosy-Westphal et al. (15); TBW, total body water; ECW, extracellular water; IW, intracellular water; BCM, body cell mass*.

### Standardized PhA and Nutrition Evaluation

**Figure 1:**
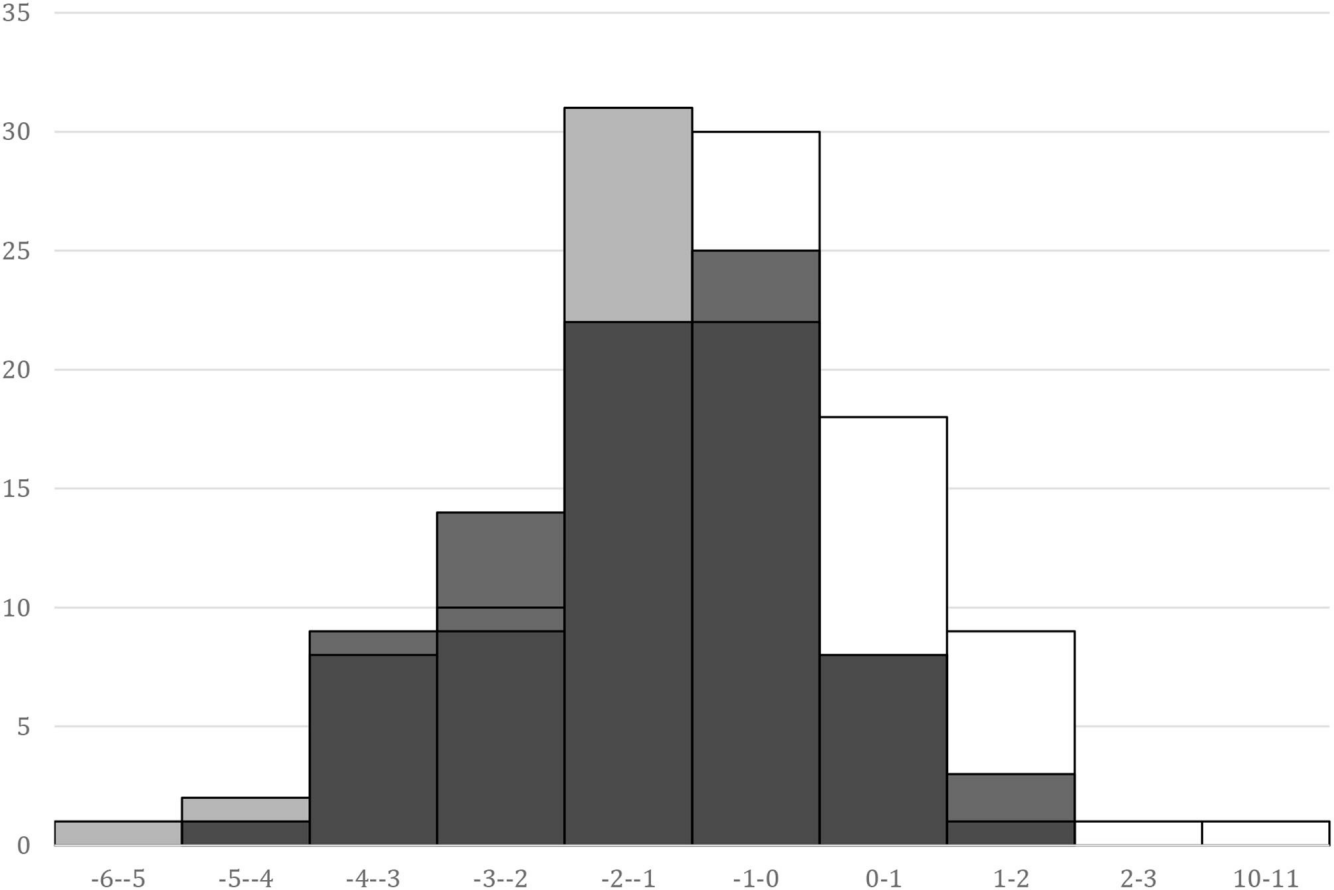
SPhA histograms. “basal,” white – SPhA before kidney transplantation; “dis,” gray – SPhA at the hospital discharge; “6 mo,” black – SPhA after 6 months after kidney transplantation.

The overlap of SPhA histograms at the baseline, at the discharge day from hospital, and 6 months after kidney transplant is presented in Figure 1.

The subjects with lower than median (<-1.46) SPhA_dis_ displayed lower values for the following variables 6 months after kidney transplant: plasma albumin concentration (43.4 vs. 45.1 g/L, *p* = 0.010), hemoglobin (124 vs. 133 g/L, *p* = 0.016), GNRI (113 vs. 118, *p* = 0.041), and handgrip strength (30 vs. 33 kg, *p* = 0.043). Conversely, these patients had higher ferritin concentrations (420 vs. 258 mkmoL/L, *p* = 0.026), longer inpatient stay (32 vs. 21 days, *p* < 0.001), and higher MIS scores (3 vs. 1, *p* = 0.001). The SGA nutrition questionnaire could not reveal these differences.

**Figure 2:**
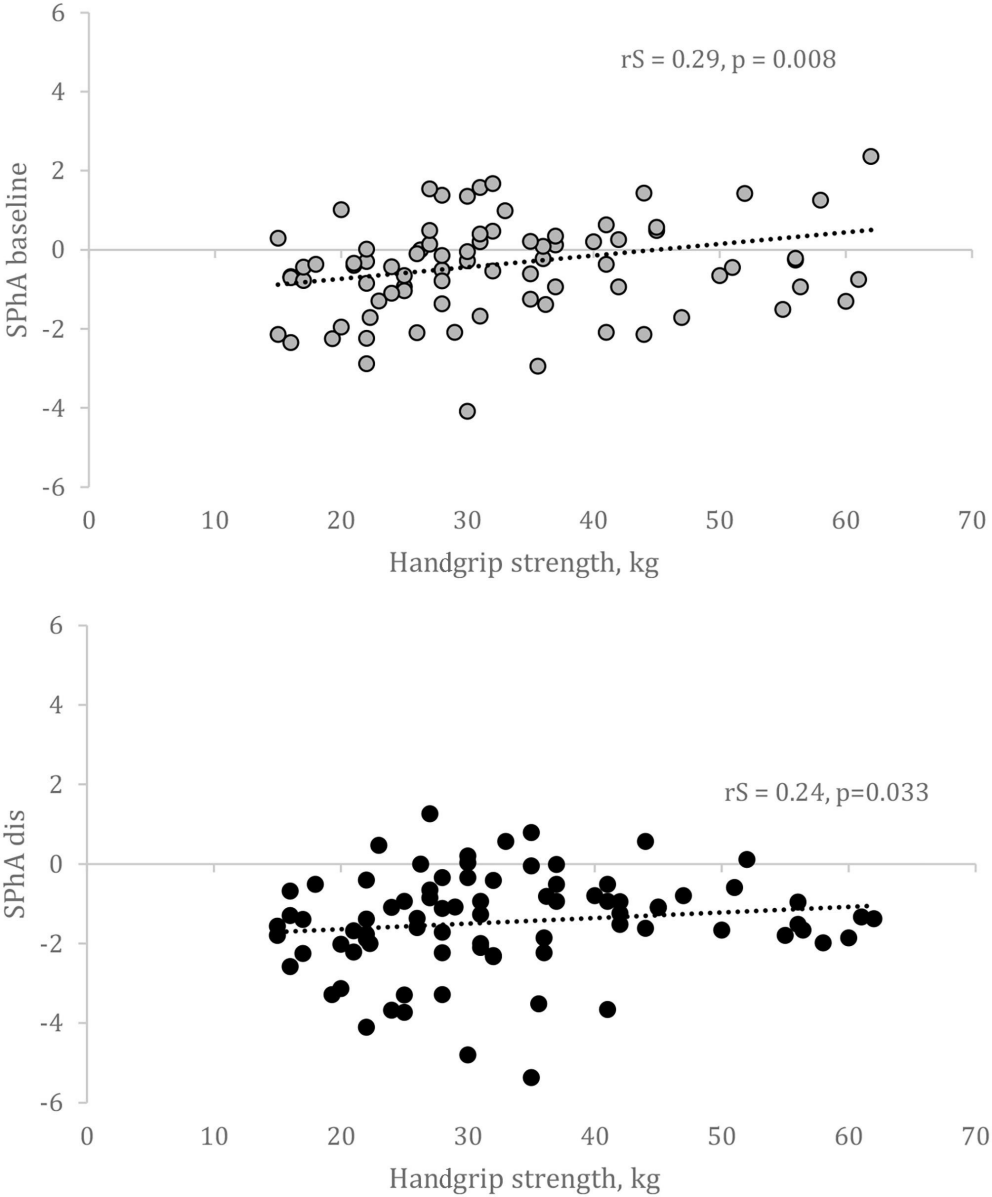
Correlation between SPhA and HGS after 6 months after kidney transplantation. SPhA – standardized PhA. rS – Spearman's correlation coefficient.

We analyzed whether the calculation of SPhA values before kidney transplant and at the hospital discharge could predict HGS 6 months after kidney transplant. The scatterplots on the association between HGS and SPhA are visualized in Figure 2.

Multivariate linear regression revealed that only in women, baseline SPhA could predict HGS following adjustments for age and baseline HGS (Table 3). We failed to confirm this association in men. Besides, in our population, HGS was not associated with dialysis vintage or other baseline variables—except for age and raw PhA values—and therefore, we did not adjust for that in the linear regression analysis.

**Table 3:** Multivariate linear regression analysis with HGS following adjustments for age and HGS before kidney transplantation.

	**SPhA at baseline**			**SPhA at discharge**		
	**Beta**	**SE**	***p*-value**	**Beta**	**SE**	***p*-value**
Handgrip strength, women	0.953	0.451	0.042	0.451	0.757	0.556

## Discussion

This study aimed to evaluate whether early posttransplant standardized phase angle (SPhA) determines nutritional status regarding different nutrition markers and predicts HGS 6 months after kidney transplantation. The main finding of this study indicates that *lower* than the median SPhA at the hospital discharge is related to protein-energy wasting, represented as lower concentrations of nutrition biomarkers (measured by plasma albumin, hemoglobin), an active inflammatory response (ferritin, MIS), whereas *higher* SPhA before kidney transplantation predicts HGS 6 months after kidney transplantation. In addition, this finding is specific to women.

One potential explanation of our results could be the longer hospital stay of the patients with lower SPhA caused by surgical and/or infectious complications (16). Furthermore, the underlying CKD-related and posttransplant inflammatory responses observed as increased ferritin—acute phase protein—levels and higher MIS scores reflected on impaired nutritional status. Ringaitiene et al. demonstrated the associations among the low PhA, low muscle mass, and decreased HGS in cardiac patients (17). In the setting of heart failure, low PhA is associated with increased mortality and prolonged length of stay (18). Patients with a lower PhA also had a higher risk of complications after surgical procedures (19). Kosoku A et al. showed that PhA was negatively correlated with sarcopenia in kidney transplant recipients (5). However, dos Reis et al. reported that PhA was associated with only HGS but not with other sarcopenia components or sarcopenia in kidney transplant recipients (20). Notably, the latter studies used unstandardized PhA values.

In line with previous studies, a low PhA is related to lower HGS, a criterion for diagnosing sarcopenia, which results from loss of SMM and strength (20). We also show that higher unadjusted SPhA is associated with higher HGS 6 months after transplantation, irrespective of sex. However, adjusted SPhA predicts HGS only in women. PhA has previously been reported to correlate with biomarkers of muscle degeneration and to decrease after muscle injury (21, 22). This supports the relationship between decreased PhA and not only low muscle mass but also impaired muscle function (23).

Passadakis et al. found that in continuous ambulatory peritoneal dialysis patients, PhA was only significantly different between well-nourished and severely malnourished groups, with a weak correlation between SGA and PhA (24). Varan et al. showed a poor specificity of a low PhA for predicting malnutrition in geriatric patients (7). The possible explanations of a poor specificity could be the use of unstandardized PhA and nutritional risk assessment with the Nutritional Risk Screening Tool 2002 (NRS-2002). In contrast, we found a significant association among standardized PhA, MIS, and hypoalbuminemia but not SGA in our study. Even though SGA has been considered as a tool applicable for patients with CKD, there are many studies claiming otherwise. They show that MIS that includes 7 SGA components and TIBC, albumin and BMI, and GNRI is superior over SGA in malnutrition evaluation (25–27).

Kidney transplantation, as a major surgical procedure, causes protein catabolism, which leads to the loss of muscle tissue, decreased albumin concentration, and impaired nutritional status. However, the use of immunosuppressive therapy and often abnormal renal function can affect the body composition of renal recipients, particularly causing the loss of muscle mass. In general, an increase in body weight is observed after renal transplantation, but at least initial posttransplant weight gain appears to be predominant due to an increase in fat mass (28). Therefore, it is important to start systematic and supervised physiotherapy immediately after surgery in combination with dietary measures.

For example, patients with a lower functional capacity at discharge are at risk of maintaining posttransplant physical inactivity (29). Both aerobic training and resistance training interventions appear to be clinically beneficial in kidney transplant recipients (30). Even though kidney transplant recipients increase their posttransplant physical activity level, they do not reach the same level as their age-matched healthy controls based on remaining compromised functional capacity, due to the combined effects of prior deconditioning, uremic myopathy, and muscle atrophy, which are also partly exacerbated by immunosuppressive and steroid therapy (31). The proven benefits of regular physical activity include healthy bones, muscles, and joints and reduced risk of premature mortality from arteriosclerotic disease and hypertension (32). This study encouraged us to launch an early posttransplant physiotherapy or rehabilitation program at our hospital and to prove the need for organizational measures to improve the ambulatory stage of physical rehabilitation and psychological therapy. Each patient should feel encouraged to participate in exercise training adjusted for individual capacity.

A major advantage is that we analyzed not raw but standardized PhA values to detect the relationship between PhA and nutrition status during the follow-up. To our knowledge, this study is the first that has investigated the role of SPhA in the assessment of nutritional status in the very early period after kidney transplantation.

The main limitations of this study are the single-center design, the small sample size, and the absence of CT/MRI data for sarcopenia evaluation.

## Conclusions or Future Directions

In summary, our study shows the predictive potential of *lower* than the median standardized PhA regarding PEW and an active inflammatory response, whereas *higher* SphA prognosticate HGS within 6 months following transplant. There is an emerging need for standardized PhA values, not specific to the general population but to CKD patients.

Future studies may examine the utility of PhA alone or in combination with other prognostic tools and how PhA may inform prognosis-based clinical decision-making in the kidney transplant setting.

## Data Availability Statement

The raw data supporting the conclusions of this article will be made available by the authors, without undue reservation.

## Ethics Statement

The studies involving human participants were reviewed and approved by Lithuanian Bioethics Committee. The patients/participants provided their written informed consent to participate in this study.

## Author Contributions

DS, MM, and LR were responsible for study design, data collection, and wrote the paper. DS performed the statistical analysis and analyzed data. All authors critically reviewed the manuscript and approved the final draft.

## Conflict of Interest

The authors declare that the research was conducted in the absence of any commercial or financial relationships that could be construed as a potential conflict of interest.

## Publisher's Note

All claims expressed in this article are solely those of the authors and do not necessarily represent those of their affiliated organizations, or those of the publisher, the editors and the reviewers. Any product that may be evaluated in this article, or claim that may be made by its manufacturer, is not guaranteed or endorsed by the publisher.
