# Supplementary material for: Standardized Phase Angle for Predicting Nutritional Status of Hemodialysis Patients in the Early Period After Deceased Donor Kidney Transplantation
Source: Front Nutr. 2022 Feb 16;9:803002. doi: 10.3389/fnut.2022.803002 (PMC8889040; doi:10.3389/fnut.2022.803002)
Supplement: Supplementary file 1 [file Data_Sheet_1.docx]

**Supplemental material.**

Phase angle and standardized phase angle values for each study patient.

| Patient | Sex | PhA°  "basal" | SPhA° "basal" | PhA°  "dis" | SPhA° "dis" | PhA°  "6 mo" | SPhA°  "6 mo" |
| --- | --- | --- | --- | --- | --- | --- | --- |
| 1. | F | 4.2 | -2.25 | 3.3 | -3.28 | 3.5 | -3.28 |
| 2. | F | 5.9 | 0.25 | 4.3 | -2.10 | 4.8 | -1.37 |
| 3. | F | 5.5 | -0.79 | 5.4 | -0.94 | 5.4 | -0.94 |
| 4. | M | 5 | -2.09 | 5.7 | -1.09 | 5.1 | -1.94 |
| 5. | F | 5.2 | -0.40 | 4.2 | -1.68 | 4 | -1.94 |
| 6. | F | 4.8 | -1.37 | 3.5 | -3.28 | 3.5 | -3.28 |
| 7. | F | 4.7 | -1.04 | 2.6 | -3.73 | 4 | -1.94 |
| 8. | F | 5.4 | -0.85 | 4.7 | -1.88 | 4.7 | -1.88 |
| 9. | M | 6.2 | -0.37 | 5.8 | -0.94 | 6.4 | -0.09 |
| 10. | F | 3.9 | -2.35 | 3.5 | -2.58 | 3 | -3.22 |
| 11. | M | 5 | -2.09 | 3.9 | -3.66 | 4.2 | -3.23 |
| 12. | M | 6.3 | -0.51 | 5.1 | -2.23 | 5.2 | -2.09 |
| 13. | F | 5.1 | -0.68 | 5.1 | -0.68 | 3.6 | -2.76 |
| 14. | M | 6 | -1.00 | 4.4 | -2.94 | 4 | -3.51 |
| 15. | M | 5.8 | -0.94 | 5.4 | -1.51 | 4.2 | -3.23 |
| 16. | M | 6.3 | -0.23 | 4.9 | -2.23 | 6.5 | 0.06 |
| 17. | F | 6.3 | 0.47 | 5.7 | -0.41 | 5.9 | -0.12 |
| 18. | M | 6.7 | -0.26 | 5.8 | -1.51 | 5.9 | -1.38 |
| 19. | F | 5.9 | -0.29 | 4.9 | -1.76 | 4.6 | -2.32 |
| 20. | M | 5.5 | -1.71 | 5.9 | -0.80 | 4.9 | -2.23 |
| 21. | F | 5.5 | -0.88 | 4 | -3.12 | 4.9 | -1.78 |
| 22. | F | 5.8 | -0.14 | 4.7 | -1.71 | 4.5 | -2.00 |
| 23. | M | 5.8 | -1.51 | 5.6 | -1.79 | 6.3 | -0.82 |
| 24. | M | 8.7 | 2.36 | 5.7 | -1.37 | 6.8 | 0.20 |
| 25. | M | 6.2 | -0.66 | 5.5 | -1.66 | 5.1 | -2.23 |
| 26. | M | 7 | 0.49 | 5.9 | -1.09 | 6.2 | -0.66 |
| 27. | M | 4.4 | -2.15 | 4.8 | -1.61 | 3.9 | -2.81 |
| 28. | F | 5.9 | -0.28 | 6.1 | 0.21 | 5.4 | -0.82 |
| 29. | M | 3.6 | -4.09 | 3.1 | -4.80 | 4.4 | -2.94 |
| 30. | M | 7.9 | 1.42 | 7 | 0.12 | 6.9 | -0.03 |
| 31. | F | 5.3 | -0.35 | 3.9 | -2.21 | 4.2 | -1.95 |
| 32. | F | 13.1 | 10.34 | 5.2 | -1.24 | 5.7 | -0.49 |
| 33. | M | 7.7 | 1.43 | 7.1 | 0.57 | 6.5 | -0.29 |
| 34. | M | 6.5 | 0.13 | 6.4 | -0.01 | 6.2 | -0.29 |
| 35. | F | 5.7 | -0.66 | 3.9 | -3.29 | 4.5 | -2.37 |
| 36. | M | 6.2 | 0.25 | 5.3 | -0.95 | 5.4 | -0.83 |
| 37. | M | 6 | -0.61 | 6.4 | -0.04 | 6.5 | 0.10 |
| 38. | M | 6.6 | -0.22 | 6.1 | -0.96 | 5.3 | -2.13 |
| 39. | M | 6.4 | -0.75 | 6 | -1.33 | 6.3 | -0.90 |
| 40. | M | 5.9 | -1.25 | 3.1 | -5.37 | 4.7 | -3.01 |
| 41. | M | 6 | -1.31 | 5.6 | -1.86 | 6.4 | -0.75 |
| 42. | F | 4.7 | -1.71 | 4.5 | -2.00 | 4.2 | -2.35 |
| 43. | F | 4.3 | -2.24 | 3 | -4.10 | 3.6 | -3.29 |
| 44. | M | 6.7 | -0.45 | 6.6 | -0.59 | 6.6 | -0.41 |
| 45. | F | 4.1 | -2.88 | 5.1 | -1.39 | 4.4 | -2.43 |
| 46. | F | 5.5 | -0.71 | 5.1 | -1.29 | 4.9 | -1.59 |
| 47. | F | 5.2 | -0.78 | 4.2 | -2.25 | 4.6 | -1.66 |
| 48. | F | 5.1 | -1.29 | 6.3 | 0.47 | 7 | 1.50 |
| 49. | F | 6.9 | 1.35 | 6 | 0.03 | 5.5 | -0.71 |
| 50. | F | 4.4 | -1.96 | 3.6 | -3.13 | 4.2 | -2.25 |
| 51. | F | 5.5 | -0.79 | 5.8 | -0.34 | 5 | -1.54 |
| 52. | F | 4.5 | -2.15 | 4.9 | -1.56 | 4.6 | -2.00 |
| 53. | M | 6.8 | 0.20 | 6 | -0.94 | 5.8 | -1.23 |
| 54. | M | 6.9 | 0.01 | 6.6 | -0.40 | 6.9 | -0.17 |
| 55. | M | 6.5 | 0.39 | 5.4 | -1.27 | 5.3 | -1.42 |
| 56. | M | 6 | -0.94 | 6.3 | -0.51 | 6.2 | -0.66 |
| 57. | F | 5.3 | -0.44 | 4.6 | -1.40 | 3.2 | -3.32 |
| 58. | M | 7.3 | 0.57 | 6.1 | -1.10 | 6.7 | -0.26 |
| 59. | F | 5.7 | -0.04 | 5.5 | -0.34 | 4.9 | -1.22 |
| 60. | M | 7.1 | 0.63 | 6.3 | -0.51 | 6.6 | -0.09 |
| 61. | M | 7.4 | 1.38 | 5.5 | -1.12 | 5.3 | -1.42 |
| 62. | M | 6.4 | -0.37 | 6.3 | -0.51 | 5.6 | -1.51 |
| 63. | M | 6.7 | 0.34 | 5.8 | -0.94 | 5.7 | -1.09 |
| 64. | M | 6.6 | 0.20 | 5.9 | -0.80 | 5.8 | -0.94 |
| 65. | F | 5.7 | -0.24 | 4.9 | -1.39 | 5.3 | -0.81 |
| 66. | M | 7.6 | 0.99 | 7.3 | 0.57 | 7.2 | 0.43 |
| 67. | M | 7.1 | 0.14 | 7.8 | 1.26 | 7.6 | 0.99 |
| 68. | M | 5.2 | -1.68 | 4.9 | -2.10 | 4.7 | -2.38 |
| 69. | M | 5.7 | -0.43 | 5.2 | -1.09 | 5.4 | -0.81 |
| 70. | M | 7.6 | 1.25 | 5.4 | -1.99 | 4.7 | -3.01 |
| 71. | M | 7 | 0.49 | 6.2 | -0.66 | 5.6 | -1.51 |
| 72. | F | 7.2 | 1.54 | 5.6 | -0.85 | 5.9 | -0.52 |
| 73. | M | 7.8 | 1.57 | 5.3 | -2.00 | 5.8 | -1.29 |
| 74. | M | 6.8 | 1.01 | 4.5 | -2.01 | 5.4 | -0.83 |
| 75. | M | 7.6 | 1.67 | 4.8 | -2.33 | 6 | -0.61 |
| 76. | M | 7 | 0.08 | 5.6 | -1.86 | 6 | -1.31 |
| 77. | M | 5.5 | -0.54 | 4 | -2.31 | 3.8 | -2.54 |
| 78. | M | 7.1 | 0.91 | 5.8 | -0.94 | 5.3 | -1.66 |
| 79. | F | 6.1 | 0.21 | 6.5 | 0.79 | 5.8 | -0.24 |
| 80. | F | 5.1 | -1.10 | 3.3 | -3.67 | 2.7 | -4.53 |
| 81. | F | 5.8 | 0.29 | 4.3 | -1.79 | 4.2 | -1.68 |
| 82. | F | 6.1 | -0.10 | 5.1 | -1.60 | 5.9 | -0.40 |

M – male, F – female, PhA – phase angle, SPhA - standardized phase angle, “basal” – before kidney transplantation, “dis” – at the hospital discharge, “6 mo” – 6 months after kidney transplantation.
